# Ethics and Invertebrates: The Problem Is Us

**DOI:** 10.3390/ani13182827

**Published:** 2023-09-06

**Authors:** Jennifer A. Mather

**Affiliations:** Department of Psychology, University of Lethbridge, Lethbridge, AB T1K 3M4, Canada; mather@uleth.ca

## Abstract

**Simple Summary:**

Why do we need a collection of papers about the welfare of invertebrates, and what do we need to learn from it? The most important reason is that they make up most of the animals on the planet, so animal welfare without invertebrates simply is not the welfare of animals. Being vertebrates and mammals, we have concentrated on knowing about animals like us, and so there is a huge lack of information about invertebrates. We cannot care about them if we do not know they exist. More than that, we do not particularly like them. They are often small, seen as ugly cockroaches, parasitic ticks, biting mosquitoes, but think instead of dazzling butterflies, flexible octopuses, attractive fireflies. Most of all, we do not think about their welfare because we see them as just machines, no complex behavior and intelligence, not deserving of care. Recent science research is changing that, and we are beginning to see that they are often very smart and sensitive and deserve our consideration. Why this is true and how we can extend welfare to non-vertebrates is the subject of this set of papers.

**Abstract:**

In the last few decades, science has begun to make great strides at understanding how varied, fascinating, and intelligent invertebrate animals are. Because they are poorly known, the invertebrates that make up about 98% of the animals on the planet have been overlooked. Because they are seen as both simple and unattractive, children and their teachers, as well as the general public, do not think they need care. Because until recently we did not know they can be both intelligent and sensitive—bees can learn from each other, butterflies can navigate huge distances, octopuses are smart, and lobsters can feel pain—we have to give them the consideration they deserve. This collection of papers should help us to see how the lives of invertebrates are tightly linked to ours, how they live, and what they need in terms of our consideration and care.

## 1. Introduction

Why does the situation of invertebrate welfare start with us? Because the biggest problems with invertebrate welfare are centered around humans, not the animals themselves. We have come to dominate the planet during the present epoch, now deservedly called the Anthropocene [1]. The damage we have done to earth and the animals on it is likely caused by the early Western philosophical approach to these animals, starting with the *scala naturae*, the belief in ascending improvement leading to humans as the peak of evolution [2]. This viewpoint was picked up by the Christian church with its belief that ‘man shall have dominion’, and due to Descartes’ assertion that animals were mere things to be manipulated, distinct from humans who had souls.

Of course, this is not the only ethical approach to animals. Buddhists believe in the brotherhood of all animals and the transformation of living things after death, along with the continuity of energy [3]. Additionally, the plains Indians believed in the one-ness of nature and our brotherhood with animals [4]. But, the Christian view became the dominant one in science. This anthropocentrism caused three problems for invertebrates: a lack of knowledge, negative attitudes, and a misunderstanding of their cognitive abilities, all leading to problems for welfare consideration. These played out through the interacting trio of (1) *scientific knowledge*, (2) *beliefs of the general public*, and (3) *political/commercial interests*.

## 2. We Do Not Know Much about Invertebrate Animals

In terms of the scientific lack of knowledge about invertebrates, Schleffers et al. [5] discuss what the term ‘missing biodiversity’ means. Mammals comprise 0.5% of the animal species on the planet, and yet we have named and identified 97% of them, but probably only half of the molluscs and one third of the crustaceans. This is particularly true of animals in unstudied ecosystems such as oceans, which make up 70% of the habitable space on the planet. This lack of information has led to the decade-long *Census of Marine Life,* a major attempt by science to find some of these animals. It has been a huge advance, but we are still catching up. This gap in information is partly the result of neglect by scientists, as an examination of recent papers in the journal *Animal Behaviour* [6] showed that 1/3 of papers were on these mammals and only 21% were on insects, who represent 170 times as many species (1). Further, papers in leading animal welfare journals have focused nearly completely on care for mammals. The result is that legislation protecting the welfare of invertebrates in research exists in only a few countries in the world, particularly in the UK, European Union, and almost only for cephalopods [6], with the United States as a notable exception.

The lack of scientific knowledge is reflected in that of the general public. Kellert [7] found that the American public knew little about invertebrates, answering only 1/3 of simple questions about them correctly. Others [8] looked at French students in Environmental and Human Sciences and found that mammals were much better known and that insects were not even seen as part of the environment. Golick [9] checked university students in the US for mistakes about insects and found them frequent. Since Kellert [7] had found that those with more education in general had a better knowledge of animals, this lack of understanding by university students makes the [9] study even more worrying.

Media feed the vertebrate centrism in our information about animals, and the public responds. Scientists have to know about animals, but they also have to feed into and inform the rest of us. The Paris Zoological Park had an ‘adoption’ program for the public to specifically target individual animals with donations. Of the 29 possible species, 20 were mammals and only one, the tarantula, was an invertebrate [10]. A similar adoption program at the Vancouver Aquarium featured three animals, two mammals and a shark, even though there are 130 marine mammals and 200,000 identified fish species. No charismatic invertebrates were featured. Albert et al. [11] performed a wide survey of the media using both an internet survey and data from aquariums and Disney and Pixar films, and found that 20 species were emphasized in these media, 17 of which were large mammals. Tourists visiting sub-Saharan Africa have been keyed to look for the ‘big five’ large mammals and their conservation is thought to support the preservation of the ecosystems of the region [12], though this excuse is only partly true. Only some of the ecosystems on the continent are preserved from human expansion and exploitation. Even when other authors [13] have explicitly said that they wanted to study anthropomorphism in the evaluation of animals, they managed to only include three invertebrates in their total of 30 species. Their view did not widen very much.

## 3. We Do Not Like or Value the Invertebrates That We Do Know about

Given this lack of information about invertebrates, perhaps it is not surprising that people have negative evaluations of them, especially of insects. They are seen as ugly, crunchy/squishy, even menacing. In fact, Schlegel et al. [14] found that despite this unpleasant view, children who could recognize them had less negative attitudes to invertebrates, so it is partly due to ignorance. This was the same with Yugoslavian university students, who had mixed views of insects, despite the fact that some of them had just taken a course on bee biology [15]. Kellert [7] pioneered the study of attitudes to animals by interviewing 29 Americans, and he found the invertebrates (examples of which were cockroach, mosquito, and wasp) occupied three of the four ‘least liked’ positions, accompanied by the rat as third-least, with affective views of fear and disgust. Given the relationship of these species to humans, this is perhaps not surprising; he could have instead chosen butterflies, dragonflies, and shrimp, for instance. A later author [16] looked at attitudes to non-human animals in terms of positions sorted on different dimensions and found four clusters in how we catalogue them: food species, popular species, intelligent ones, and dangerous ones. This could have been followed up to evaluate the attitudes to members of the different groups but it was not. We could feature ‘popular’ invertebrates such as butterflies, but we mostly do not. The negative attitudes start with children, as Swiss children showed disdain for land invertebrates [17], though they were more positive to butterflies and dragonflies [14]. In fact, butterflies were admired by residents and tourists in northern India [18] and noticed by visitors to urban parks in East and Southeast Asia [19]. Breuer et al. [17] specifically studied them as ‘flagship’ species for invertebrates in Beverin Nature Park in Switzerland, aiming to help turn attitudes around. The more general disdain can start early, as [20] found this preference in Italian kindergarten children. However, this is not surprising as both [21,22] found them showing fear and disgust toward invertebrates, and especially toward spiders.

It is not surprising that children have negative attitudes to invertebrates when you look at the values of their teachers. An evaluation of students in teacher education in Norway and Italy showed that there were negative attitudes toward invertebrates, especially in Italy and much more in females [23]. This gender gap is worrying, as early education teachers are predominantly female, and prejudices learned early are hard to counter. Still, the cultural difference shows that these negative attitudes are learned and not innate. This is particularly true as ‘outdoor education’ is a feature of Norwegian life and children meet and interact much more with invertebrates than they would if they were indoors playing on the computer. The dislike and ignorance are not just European, as there is a low level of knowledge and understanding of invertebrates in Indonesia too [24]. A short experience is not always enough to change sweeping attitudes, as exposure to Madagascar cockroaches changed pre-teacher attitudes to that insect but did not generalize to other species [22].

However, it is relatively easy to turn this ignorance and dislike around somewhat in children. German school children’s visits to a ‘green classroom’ outside the school had a long-term effect on them noticing that invertebrates existed [25]. This was shown when they drew pictures of what lived there, as they now especially noticed the soil and its inhabitants. Japanese gardeners had biophilia (liking) to insects and not the more common biophobia (aversion) found in their society [26], again having experienced the ecosystem ‘close up’. Many American elementary schools have gardens in the schoolyard for children to work in, a break from, and extension of, the classroom. This began with the goal of increasing their likelihood of eating a healthier diet, especially including vegetables [27], assuming that the involvement and good taste would win them over. Tending to the soil seems to have a mental health outcome as well, with the change in focus and the productivity helping with stress. However, given the Japanese study, it may increase children’s knowledge of, and comfort with, invertebrates as a side effect.

## 4. We Do Not Know How Smart and Sensitive They Are

Because of our anthropocentric view of animals, we have long underestimated the intelligence of any invertebrate group. The dominant belief in the evolution of cognitive ability is the social intelligence hypothesis [28,29]. Focusing particularly on primates, researchers have argued that the pressure to understand other individuals in tight-knit social groups has led to the flexibility of actions and knowledge of consequences. They particularly talked about the ‘theory of mind’, the necessity to understand the motivation of others. The mammalian, and especially primate, combination of big brains, long life, parental care, and these complex social groups have fostered learning and its use. Still, the evaluation of hyenas argued for a modified version [30], and others saw a combined ecological-social one [31], but the paradigm was fairly fixed, dominated the study of animal cognition, and generally eliminated invertebrates from any considerations of intelligence, sentience, and thus welfare.

As a result of this view, several biases have interfered with a fair assessment of invertebrate abilities and their need for welfare consideration [32]. One is the belief that different structures and systems cannot be convergent for mental operations. Mammals have a cortex and consciousness, so no cortex means no consciousness. A second is that size (big brains) is a dominant influence and small brains cannot carry out the complex operations needed for intelligence. A third is the mammal-centered use of testing animals for cognitive ability within situations in the sensory modality and adaptation for the structures of vertebrates. The ‘mark test’ of Gallup [33], demanding visual dominance and non-living skin structure such as fur, is the most obvious. Finally, there is the excessive use of Morgan’s canon, the belief that any simple explanation of the source of behaviour must be the correct one if it is held up against a more complex one. The answer is ‘usually but not always’.

As more evidence accumulated about the intelligence of cephalopod molluscs in particular [34,35], it became apparent that these invertebrates were very intelligent. This was brought to the more general attention of scientists by the Cambridge Declaration of Consciousness [36]. A gathering of leading neuroscientists and behaviorists evaluated animals with the aim to find possible neural substrates for consciousness. Among other groups, octopuses were suggested as candidates for this ability, and this judgment was widely circulated. A philosopher’s semi-popular book about cephalopods’ cognitive ability [37] reached many in the scientific community, and a more subjective and popular one about the author’s experiences with them [38] in the ocean and in captivity was read by the general public. Craig Foster’s documentary *My Octopus Teacher* [39], besides winning an Oscar, reached all across the globe, and gradually resulted in a huge expansion of public information and sympathy for them, thus slowly changing people’s attitudes. Popular opinion about how the intellectual ability of cephalopods could have evolved is still varied, leading a group of cosmologists to ignore the biological evidence and suggest that octopuses were aliens who had actually descended from space as cryopreserved eggs [40].

The scientific presentation of the possibility of insect consciousness [41] has fared less well. The authors take a neurobiological approach to proof of consciousness and suggest that the seat of vertebrate consciousness may be not the cortex but the midbrain, allowing them to evaluate insect consciousness ‘on a more level playing field’. As they expected when they produced these ideas, there were what they called “lively areas of disagreement”. As Michalevich and Powell [32] suggested, opponents to the consideration of insects as intellectual advanced the size problem, disbelieved the analogy, and made the call for retreat to simplicity in explanations. Additionally, there was much discussion of the relative roles of the cortex and midbrain in consciousness. Research continues to ‘chip away’ at the exclusivity of complex behaviour as a mammalian domain, and the disdain for insects’ cognitive competence. See [42] for insect navigation, [43] for social cognition, and [44] for a suggestion of a direct comparison between insects and vertebrates; however, changing a paradigm is slow work. In terms of the public presentation of this scientific information, the recently published short book Mind of a Bee [45] is aimed at a wider audience. It is a start, but further developments will be needed to convince the public.

This collection of papers will be an introduction to why and how we should ‘do’ invertebrate welfare. It is not directed specifically at the general public, but at those seeking education on this wider view of animal welfare. There is a complex web of scientists, public opinions, and institutions. This includes ‘farmers’ who raise domestic species, governments who regulate what we can and must do to invertebrates, and NGOs who advocate for animals. But here is where and with whom it starts.
